# Noninvasive Assessment of Liver Fibrosis: Current and Future Clinical and Molecular Perspectives

**DOI:** 10.3390/ijms21144906

**Published:** 2020-07-11

**Authors:** Ryota Masuzaki, Tatsuo Kanda, Reina Sasaki, Naoki Matsumoto, Masahiro Ogawa, Shunichi Matsuoka, Seth J. Karp, Mitsuhiko Moriyama

**Affiliations:** 1 Division of Gastroenterology and Hepatology, Department of Medicine, Nihon University School of Medicine, Itabashi-Ku, Tokyo 173-8610, Japan; kanda.tatsuo@nihon-u.ac.jp (T.K.); sasaki.reina@nihon-u.ac.jp (R.S.); matsumoto.naoki@nihon-u.ac.jp (N.M.); ogawa.masahiro@nihon-u.ac.jp (M.O.); matsuoka.shunichi@nihon-u.ac.jp (S.M.); moriyama.mitsuhiko@nihon-u.ac.jp (M.M.); 2 Division of Liver Transplantation, Department of Surgery, Vanderbilt University Medical Center, Nashville, TN 37232, USA; seth.karp@vanderbilt.edu

**Keywords:** hepatocellular carcinoma, risk factor, liver fibrosis, elastography, serum marker, extracellular matrix, matrix metalloproteinase, tissue inhibitor of metalloproteinase

## Abstract

Liver fibrosis is one of the risk factors for hepatocellular carcinoma (HCC) development. The staging of liver fibrosis can be evaluated only via a liver biopsy, which is an invasive procedure. Noninvasive methods for the diagnosis of liver fibrosis can be divided into morphological tests such as elastography and serum biochemical tests. Transient elastography is reported to have excellent performance in the diagnosis of liver fibrosis and has been accepted as a useful tool for the prediction of HCC development and other clinical outcomes. Two-dimensional shear wave elastography is a new technique and provides a real-time stiffness image. Serum fibrosis markers have been studied based on the mechanism of fibrogenesis and fibrolysis. In the healthy liver, homeostasis of the extracellular matrix is maintained directly by enzymes called matrix metalloproteinases (MMPs) and their specific inhibitors, tissue inhibitors of metalloproteinases (TIMPs). MMPs and TIMPs could be useful serum biomarkers for liver fibrosis and promising candidates for the treatment of liver fibrosis. Further studies are required to establish liver fibrosis-specific markers based on further clinical and molecular research. In this review, we summarize noninvasive fibrosis tests and molecular mechanism of liver fibrosis in current daily clinical practice.

## 1. Introduction

Hepatocellular carcinoma (HCC), one of the most common malignancies worldwide, is usually accompanied by advanced liver fibrosis or cirrhosis [1,2]. The etiology of background liver diseases differs geographically, however, chronic viral hepatitis due to either hepatitis B virus (HBV) or hepatitis C virus (HCV) is the leading cause of HCC in many countries [3,4,5]. Other major etiologies, such as alcoholic liver disease (ALD) and nonalcoholic steatohepatitis (NASH) have also been increasing [6].

Liver fibrosis, which is a consequence of inflammation and regeneration, represents accumulated damage to DNA in hepatocytes. Indeed, liver fibrosis seems to be one of the risk factors for HCC development [7]. Until recently, the staging of liver fibrosis could only be assessed with a liver biopsy. However, liver biopsy occasionally causes severe complications in up to 3% of patients, including death in 0.03% [8]. There is, therefore, a need for an accurate noninvasive test for the diagnosis and staging of liver fibrosis.

**Figure 1:**
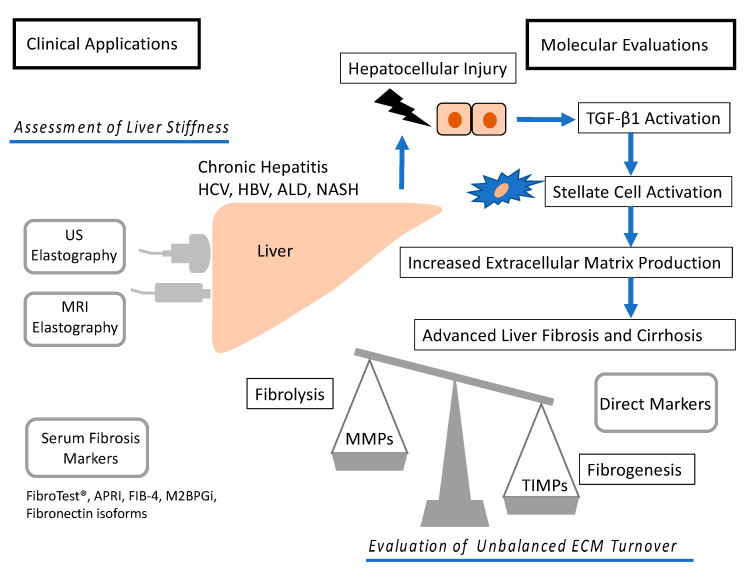
Molecular mechanism and diagnosis of development of liver fibrosis. Hepatocellular injury causes TGF-β1 activation. In turn, TGF-β1 activates hepatic stellate cell and increases ECM production, causing liver fibrosis and cirrhosis. The development is depending on the balance between fibrolysis and fibrogenesis. ECM turnover is controlled by MMPs and TIMPs. Liver fibrosis can be assessed by elastography and serum markers such as aspartate aminotransferase (AST) to platelet ratio index (APRI), FIB-4, and Mac-2 binding protein glycosylation isomer (M2BPGi). US, ultrasound; MRI, magnetic resonance imaging; HCV, hepatitis C virus; HBV, hepatitis B virus; ALD, alcoholic liver disease; NASH, nonalcoholic steatohepatitis; APRI, aspartate aminotransferase to platelet ratio index; M2BPGi, Mac-2 binding protein glycosylation isomer; MMPs, matrix metalloproteinase; TIMPs, tissue inhibitors of metalloproteinases; TGF-β1, transforming growth factor β1.

Fibrosis is accompanied by an accumulation of extracellular matrix (ECM), following the activation of hepatic stellate cells (HSCs) and the production of transforming growth factor β1 (TGF-β1). We previously reported that simple stromal injury mimics liver fibrosis with HSC activation, fibronectin production, and collagen deposition using a mouse model [9]. In a healthy liver, the turnover of ECM is regulated by enzymes called matrix metalloproteinase (MMPs) and their specific inhibitors, tissue inhibitors of metalloproteinases (TIMPs). The context of this review is demonstrated in Figure 1.

In this review, we summarize noninvasive fibrosis tests that have already been applied in daily clinical practice and review the molecular mechanism of liver fibrosis and some candidate molecular markers that are now being studied and are expected to be used in future clinical practice.

## 2. Noninvasive Imaging Techniques

Elastography is a noninvasive imaging technique and is now widely accepted as a fibrosis assessment in clinical practice. Most methods measure the propagation speed of shear waves to estimate liver stiffness. There are ultrasound (US) elastography and magnetic resonance imaging (MRI) elastography. The diagnostic performance and characteristics of each technique are listed in Table 1 and Table 2.

### 2.1. US Elastography

#### 2.1.1. Static Strain Imaging

In static strain elastography, pressure is generated by mechanical or manual compression, and then the amount of target lesion deformation is measured [29]. The applied compression is either manual by the transducer or physiological from the heartbeat or lung movements [16]. The main clinical use of this technique is in the evaluation of surface organs such as breast and thyroid lesions [17]. This technique could be potentially useful in evaluating large liver tumors: and for discriminating hard and soft tumors [18].

#### 2.1.2. One-Dimensional Transient Elastography

Transient elastography is the first commercialized shear wave elastography (SWE) system. Transient elastography has a single measuring device that contains a vibrator and an ultrasound transducer [19,20]. The new XL probe of Firboscan^®^ (Echosens, Paris, France) is now commercially available because of the frequent measurement failure of standard probes in obese patients [21]. The diagnostic performance (sensitivity, specificity, cutoff values, area under the receiver operating characteristic curve [AUROC]), as reported in a meta-analysis, is demonstrated in Table 1 [10,11]. Transient elastography is the most widely studied and accepted elastography method and it is reported to be useful in predicting clinical outcomes [22,23,24,25]. The advantages of transient elastography are its wide range value (from 0 kilopascal [kPa] to 75 kPa) and rapid and straightforward use in outpatient clinics. The disadvantages of the technique are the requirement of a special apparatus, the lack of two-dimensional grey scale imaging B-mode and real-time liver stiffness imaging, and difficulty in the measurement of patients with obesity, ascites, or narrow intercostal space.

#### 2.1.3. Point SWE

Point SWE uses an acoustic radiation force impulse (ARFI) to generate shear waves in the liver. The examiner is able to use grayscale ultrasound imaging to locate a small region of interest (ROI) in the right hepatic lobe, avoiding large vessels and the gallbladder. The diagnostic performance of SWE, as reported in a meta-analysis, is demonstrated in Table 1 [12,13]. The advantage of point shear wave elastography is that it can be performed under B-mode ultrasound, so tumors and large vessels can be avoided. The disadvantages of point shear wave elastography are a small ROI and lack of real-time stiffness imaging. Point SWE has similar diagnostic performance as one-dimensional transient elastography and can be performed during the daily ultrasound examination.

#### 2.1.4. Two-Dimensional Shear Wave Elastography

Two-dimensional (2D) SWE is the most recently introduced technique. Two-dimensional SWE uses multiple ARFIs at multiple locations. In a meta-analysis including 13 sites with 1134 patients with HCV, HBV, or nonalcoholic fatty liver disease (NAFLD), the diagnostic performance of 2D SWE for differentiating significant fibrosis (F2), severe fibrosis (F3), and cirrhosis were shown in Table 1 [14]. Two-dimensional elastography has a real-time color-coded map, and heterogeneity can also be evaluated along with the stiffness value.

### 2.2. Magnetic Resonance Imaging (MRI) Elastography

MRI elastography has the advantage of assessing the whole liver, compared to the limited assessment by US elastography or even liver biopsy. Continuous mechanical waves (60 Hz mechanical compressions) are produced from the active driver outside the examination room to the passive driver positioned on the patient’s body over the liver, resulting in periodic liver tissue displacement [26]. In a meta-analysis, including 12 studies with various etiologies [27], the diagnostic performance of MRE is as demonstrated in Table 1. It is essential to know that the measured parameters of liver stiffness are not equal between the different techniques [28]. For instance, although transient elastography and MRE have different mechanisms and different thresholds for the diagnosis of cirrhosis, both measurements are expressed in kPa. Since measurement failure for obese patients is common in ultrasound-based elastography, MRI elastography could be especially useful in those obese nonalcoholic fatty liver disease (NAFLD) patients.

### 2.3. Noninvasive Biomarkers and Their Combinations

Liver function tests are routinely used for the management of all chronic liver diseases. AST, alanine aminotransferase (ALT), gamma-glutamyl transferase (GGT), total bilirubin, albumin, prothrombin time (PT), and platelet count are routinely checked in out-patient clinics. Noninvasive biomarkers can be applied together with the routine blood draw. The features and diagnostic performance of each test are shown in Table 3.

#### 2.3.1. FibroTest^®^

FibroTest^®^ (Biopredictive, Paris, France) (FT) is a biomarker of liver fibrosis that was initially reported and validated in patients with chronic HCV infection [29]. This test includes α2-macroglobulin, haptoglobin, GGT, γ-globulin, total bilirubin, and apolipoprotein A1. In a meta-analysis including a total of 30 studies, the individual data were analyzed in 3282 patients and AUROCs are demonstrated in Table 3 [30]. FT is a commercially available test and has good performance in the diagnosis of the liver fibrosis stage.

#### 2.3.2. APRI

AST to platelet ratio index (APRI) was calculated as (AST level /upper limit of normal [ULN])/platelet counts (10^9^/L) × 100 [36]. The result can be obtained from a web-based calculator. In a meta-analysis, including 40 hepatitis C-related fibrosis studies and 16 hepatitis B-related fibrosis studies, the diagnostic performance was as shown in Table 3 [32,33]. APRI is an index obtained from general blood tests and its diagnostic performance is comparable with other serum tests.

#### 2.3.3. FIB-4 Index

The FIB-4 index was first reported and proposed by the authors of the AIDS Pegasys Ribavirin International Coinfection Trial (APRICOT) as an index that could predict the fibrosis stage in patients coinfected with human immunodeficiency virus (HIV) and HCV [33]. The index comprises age, AST, platelet count, and ALT and is calculated as (age [years] × AST [U/L])/(platelet counts [10^9^/L] × ALT^1/2^ [U/L]). In an original study and a meta-analysis including 22 HBV-related fibrosis studies, the diagnostic performance was as shown in Table 3 [34]. In a multicenter study from Japan, the modified cutoff points were reported for different ages [37]. Fibrosis progression of chronic HCV infection is slow and generally takes several decades to develop liver cirrhosis [38]. Fibrosis progression rate in chronic HCV infection is known to depend on patients’ characteristics at the onset of infection such as age, gender, alcohol consumption [39,40,41]. Patients with rapid fibrosis progression would have died young, and those with slow progression would be able to live longer [42]. Whether the fibrosis indices should include age probably depends on the disease etiology and epidemiology. The FIB-4 index is comprised of parameters readily available in daily clinical practice.

## 3. Molecular Mechanism of Fibrosis

### 3.1. Mechanism of Fibrosis

**Figure 2:**
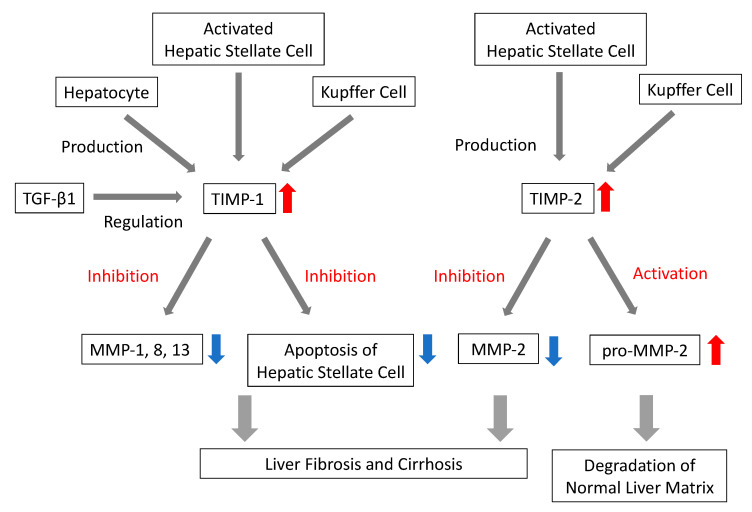
Representative mechanism of matrix metalloproteinase (MMP) and tissue inhibitor of metalloproteinase (TIMP) in liver fibrosis. Red arrows indicate activation, blue arrows indicate inhibition. TGF-β1, transforming growth factor β1. TIMP-1 is produced by activated stellate cell, hepatocyte, and Kupffer cell. TIMP-1 is regulated by TGF-β1 and inhibits collagenase (MMP-1, 8, 13) and apoptosis of hepatic stellate cell, causing liver fibrosis and cirrhosis. TIMP-2 is produced by activated stellate cell and Kupffer cell. TIMP-2 inhibits MMP-2 and also activates pro-MMP-2, causing degradation of normal liver matrix.

The mechanism of liver fibrosis has been vigorously studied, and TGF-β1 activation, stellate cell activation and deposition of ECM, and imbalance of MMPs and TIMPs are thought to be of paramount importance in fibrogenesis. One of the pathological features of liver fibrosis is the increased expression of collagens, fibronectins, proteoglycans, structural glycoproteins, and hyaluronan [43,44,45]. Fibronectin seems to play a key role in this process. On one hand, fibronectin seems to affect TGF-β release [46], and on the other hand, its production is required for the accumulation of collagen and hence fibrosis development [47,48]. Collagens are degraded by MMPs, which, together with their inhibitors, termed TIMPs, play a key role in fibrogenesis and fibrolysis [49,50,51]. These enzymes can be noninvasive fibrosis markers as they are directly involved in liver fibrosis. The family of human MMPs comprises more than 24 members and can be divided into the subgroups collagenases, gelatinases, stromelysins, matrilysins, membrane-type MMPs, and others [45,52]. Each MMP is described in the following sections based on the subgroup. Representative mechanism of MMPs and TIMPs in liver fibrosis is demonstrated in Figure 2. Summary of MMPs and TIMPs are shown in Table 4.

#### 3.1.1. Collagenase Subgroup

MMP-1, MMP-8, MMP-13, and MMP-18 are classified in this group. These enzymes can cleave interstitial collagens I, II, and III. First MMP, MMP-1 was discovered by Dr. Gross in 1962 from tadpole tissue [53]. During the metamorphosis, removal and remodeling of the tissue is precisely controlled by proteinases including MMP-1. MMP-1 plays an important role in the regression of liver fibrosis in rodents. MMP-1 degrades key collagens in hepatic fibrosis and is a promising marker for antifibrotic therapy. MMP-1 mRNA was elevated in the fibrotic and cirrhotic liver of chronic hepatitis C patients [54]. Overexpression of MMP-1 induced by human adenovirus vector expressing MMP-1 (AdMMP-1) injection attenuated liver fibrosis and stimulated hepatocyte proliferation in a rat fibrosis model [54]. The improvement after cholestatic liver injury correlated with MMP-8 activity [56]. The overexpression of MMP-8 reduced fibrosis in rat fibrosis models [57]. MMP-2, MMP-8, and MMP-9 were reported to be serum markers of disease severity in patients with alcoholic liver disease [58]. A dermal wound healing model in MMP-13 knockout mice showed decreased myofibroblast proliferation and TGF-β1 level, which indicated that MMP-13 was involved in that TGF-β1 activation [59]. MMP-13 is reported to be useful for predicting alcoholic liver cirrhosis; however, the MMP-1 levels are not significantly elevated in cirrhotic patients compared to controls [60]. The collagenase group is capable of degrading the triple helix conformation of native collagens [102].

#### 3.1.2. Gelatinase Subgroup

MMP-2 (gelatinase A) and MMP-9 (gelatinase B) are classified in this group. MMP-2 degrades type I, II, and III collagens [61,62]. MMP-2 suppresses collagen I expression [63], and the loss of MMP-2 aggravates fibrosis, suggesting that MMP-2 suppresses TIMP-1 upregulation during liver fibrosis [64]. MMP-9 promotes apoptosis of HSCs [65] and is expressed in HCC [66,67]. In a murine model, MMP-9 was used as a therapeutic target for fulminant hepatic failure, and its inhibition led to prolonged survival by improving hepatic and brain injury at an early stage [68]. The gelatinases subgroup has three repeats of a type II fibronectin domain inserted in the catalytic domain, which allows for the binding to and processing of denatured gelatin and collagens [103].

#### 3.1.3. Stromelysin Subgroup

MMP-3 (stromelysin 1) and MMP-10 (stromelysin 2) are classified in this group. Both MMPs have a similar structure; however, MMP-3 has a higher proteolytic ability than MMP-10. MMP-3 activates several pro-MMPs, and its action on pro-MMP1 seems to be important for the production of fully active MMP-1 [69]. The strong overall expression of MMP-3 and MMP-10 was found in HCCs, especially in the ECM adjacent to blood vessels [70]. Compared with healthy controls, serum samples from patients with chronic diseases had a 50% reduction in serum MMP-3 levels, as measured by enzyme-linked immunosorbent assays [71]. MMP-11 is called stromelysin 3, which plays a vital role during tumor migration, invasion, and metastasis [72,73]. The association between five single nucleotide polymorphisms (SNPs) (rs738791, rs2267029, rs738792, rs28382575, and rs131451) of the MMP-11 gene and HCC development, along with other clinical outcomes such as development of moderate to severe liver failure and distant metastasis, were reported in 293 patients with HCC and in 586 cancer-free controls [74]. The carriers of the mutant allele (CT+TT) of the rs738791 variant had a higher risk of HCC than wild-type (CC) carriers. The stromelysin group is capable of cleaving extracellular matrix proteins and its relationship with HCC is reported [74].

#### 3.1.4. Matrilysin Subgroup

MMP-7 (matrilysin 1) and MMP-26 (matrilysin 2) are classified in this group [75,76]. In addition to ECM degradation, MMP-7 processes cell surface molecules such as pro-α-defensin, Fas-ligand, pro-tumor necrosis factor (TNF)-α, and E-cadherin. The mRNA and protein level of MMP-7 is positively related to the progression of liver fibrosis in biliary atresia [76]. MMP-7 is also reported to be involved in human cancer metastases [77]. MMP-26 digests several ECM components and activates pro-MMP-9 by cleavage [79]. The common structure of MMP consists of four domains: a signal peptide to direct secretion from the cell; a propeptide maintaining enzyme latency; a catalytic domain with a Zn-binding site; and a hemopexin-like domain at the C-terminal region [51]. The common feature of the matrilysin group is that they all lack a hemopexin domain and are the smallest MMP in size.

#### 3.1.5. Membrane-Type MMP Subgroup

There are six membrane-type MMPs (MT-MMPs): four are type I transmembrane proteins (MT1-MMP [MMP-14], MT2- MMP [MMP-15], MT3-MMP [MMP-16], and MT5-MMP [MMP-24]), and two are glycosylphosphatidylinositol (GPI)-anchored proteins (MT4-MMP [MMP-17] and MT5-MMP [MMP-25]). Most MMPs are secreted in the extracellular environment, however, MT-MMPs are secreted in the plasma membrane of the producing cells, suggesting MT-MMP are essential in pericellular ECM degradation. The first MT-MMP, MT1-MMP was discovered and characterized as a cell surface proMMP-2 activator. MT1-MMP has a collagenolytic activity on type I, II, and III collagens and associated with cell invasions in malignant tumors [80]. MT1-MMP was reported to be overexpressed in highly invasive HCC with its invading border of the tumor [81]. MT1-MMPdeficient mice had severe skeletal defects possibly due to a decreased vascular invasion of calcified cartilage and it also seemed to play an important role in angiogenesis [82]. HBV X-interacting protein (HBXIP) promotes HCC cell migration and invasion through MT2-MMP. The silencing of MT2-MMP partly decreases the cell migration and invasion promoted by HBXIP overexpression [83]. MT3-MMP also promotes cell invasion and metastases [84]. MT4-MMP is reported to be expressed on the cell surface of human breast cancer cells and promotes primary tumor growth and lung metastasis [85]. MT5-MMP is brain-specific and is mainly expressed in the cerebellum and associated with neuronal development [86]. MT6-MMP is expressed predominantly in peripheral blood leukocytes, anaplastic astrocytoma, colon carcinoma cells, and glioblastoma, but not in normal colon, and meningioma [87,88].

#### 3.1.6. Other MMPs Subgroup

Six MMPs are not classified in the above-mentioned categories. MMP-12 (metalloelastase) mainly expressed in macrophages digests elastin and is reported to be associated with pulmonary fibrosis and chronic obstructive pulmonary disease [89,90]. MMP-19 was identified from a human liver cDNA library and from a synovial membrane of a patient with rheumatoid arthritis [91,92]. MMP-19 is reported to play an important role in the development of liver injury and subsequent fibrosis through influencing TGF-β1 and the insulin-like growth factor-1 (IGF-1) signaling pathway [93].

MMP-20 (enamelysin), which digests amelogenin, is primarily located within newly formed tooth enamel [94]. MMP-22 was first cloned from chicken fibroblasts, and the function of this enzyme is not known [95]. MMP-23, also called cysteine array MMP, is mainly expressed in reproductive tissues [96,97]. The latest addition to the MMP family is epilysin (MMP-28), which is mainly expressed in normal tissues, such as testis, intestine, lung, and skin. In addition, its expression patterns in injured skin suggest that MMP-28 functions in tissue hemostasis and wound repair [99,100,101]. MMP-28 promotes the epithelial to mesenchymal transition (EMT), migration, and invasion of HCC cells [101].

#### 3.1.7. TIMPs

Four TIMPs (TIMP-1, TIMP-2, TIMP-3, and TIMP-4) are known to be associated with liver fibrosis. All MMPs can be inhibited by at least one of the TIMPs. In patients with HCV, TIMP-1 serum protein and mRNA levels are positively correlated with the staging of liver fibrosis [104,105]. In situ hybridization and immunoelectron microscopy revealed TIMP-1 was localized in fibrosis septa and was possibly produced from activated HSCs [105]. Since TIMP-1 is also significantly associated with fibrogenesis in the lungs [106,107], kidneys [108,109], and pancreas [110,111], TIMP-1 seems to play a central role in tissue fibrosis. A summary of four TIMPs are shown in Table 5.

In patients infected with HCV, elevated serum protein levels and mRNA expression of TIMP-2 were reported [112,113]. In rat bile duct ligation model, the mRNA expression level of TIMP-2 was elevated after 10 days and showed no further change until 30 days [114]. A zymography study using tissue extracts revealed that TIMP-2 was necessary for activating latent pro-MMP-2 [115]. TIMP-2 also has an inhibitory function against MT1-MMP, as demonstrated in a *Timp-2* deficient mouse model [116].

TIMP-3 inhibits a disintegrin and metalloproteinase 17 (ADAM17) and its essential role in the liver was confirmed in a *Timp-3* deficient mouse model. Timp-3 deficient mice suffered necrosis, apoptosis, and morbidity after partial hepatectomy, due to the inability to downregulate hepatic TNF levels [117].

Mice lacking TIMP-4 had greater activity of MT1-MMP with increased inflammation, indicating that TIMP-4 regulates ECM deposition through MT1-MMP inhibition [118].

As shown above, TIMPs are not only the inhibitors of MMP; they have other independent biological functions, too.

#### 3.1.8. Fibronectin Isoforms

Fibrosis results from accumulation of fibronectin leading to collagen accumulation [47]. Most of the circulating fibronectin is called plasma fibronectin and lacks three characteristics that make fibronectin accumulate in the matrix. These are the presence of an EDA domain, and EDB domain or a glycosylation site leading to fibronectin being called oncofetal fibronectin. Since these isoforms are produced by the hepatic stellate cells that are responsible for matrix production [119], these isoforms were detected in patients with liver disease [120] and therefore evaluated in relationship to fibrosis and were found to predict the degree of fibrosis in chronic hepatitis C [121]. An increase in the isoform EDA over 1.32 and the isoform oFN over 3.26 in combination predicted significant fibrosis with a specificity >99%, while values below 0.78 for EDA and below 1.88 for oFN excluded significant fibrosis with a specificity of 94%. These encouraging results are probably due to the fact that the two molecules measured represent substances that directly accumulate in fibrotic tissue.

#### 3.1.9. Mac-2 Binding Protein Glycan Isomer (M2BPGi)

Fibrosis-related glycol alterations of hyperglycosylated Mac-2 binding protein (M2BP) were identified by glycan-based immunoassay and fibrosis-specific modified M2BP was termed Mac-2 binding protein glycosylation isomer (M2BPGi) [122,123]. M2BPGi was detected using a lectin called *Wisteria floribunda agglutinin* that binds specifically to M2BPGi [124]. In a meta-analysis, including 21 studies, the diagnostic performance is as shown in Table 3 [35]. Cutoff values for fibrosis stages differ among HBV- and HCV-related liver disease [125]. The difference might reflect the different mechanisms of liver fibrogenesis and should be evaluated in future studies.

## 4. Conclusions

Accurate diagnosis of liver fibrosis is essential in the management of chronic liver disease, as the fibrosis stage is regarded as a surrogate marker for evaluating the severity of the disease. US and MR elastography have become prominent as noninvasive methods for quantifying hepatic fibrosis, and they are now widely applied in clinical practice. The limitations of elastography are mainly technical challenges: the need for better-quality measurement in obese patients, threshold standardization, and cost reduction. Fibrosis seems to develop and progress as a consequence of alterations in matrix production and/or degradation, accompanied by increased matrix production. Understanding the factors that lead to increased matrix production or decreased matrix degradation will lead to new fibrosis marker development and eventually to a discovery of antifibrotic reagents. Further studies are required to establish more accurate fibrosis markers based on molecular research.

## Figures and Tables

**Table 1:** Summary of diagnostic performances of elastography reported in meta-analyses.

Elastography	Etiology		F2	F3	F4	Reference
One-dimensional Ultrasound (Transient elastography)	Various etiologies	Cutoff (kPa)	7.65	N/A	13.01	[10]
		Sensitivity	N/A	N/A	N/A	
		Specificity	N/A	N/A	N/A	
		AUROC	0.84	0.89	0.94	
	HBV	Cutoff (kPa)	7.9	8.8	11.7	[11]
		Sensitivity	74.3	74.0	84.6	
		Specificity	78.3	63.8	81.5	
		AUROC	0.859	0.887	0.929	
Point shear wave Ultrasound	Various etiologies	Cutoff (m/s)	1.31	N/A	1.80	[12]
		Sensitivity	74	N/A	87	
		Specificity	83	N/A	87	
		AUROC	0.85	N/A	0.93	
	Nonviral	Cutoff (m/s)	N/A	N/A	N/A	[13]
		Sensitivity	79	92	89	
		Specificity	81	85	89	
		AUROC	0.87	0.94		
Two dimensional Ultrasound	HCV	Cutoff (kPa)	7.1	9.2	13.0	[14]
		Sensitivity	94.7	90.3	85.8	
		Specificity	52.0	76.8	87.8	
		AUROC	0.863	0.915	0.929	
	HBV	Cutoff (kPa)	7.1	8.1	11.5	
		Sensitivity	87.6	94.9	79.9	
		Specificity	73.6	73.1	93.3	
		AUROC	0.906	0.931	0.955	
	NAFLD	Cutoff (kPa)	7.1	9.2	13.0	
		Sensitivity	93.8	93.1	75.3	
		Specificity	52.0	80.9	87.8	
		AUROC	0.855	0.928	0.917	
	Others	Cutoff (kPa)	7.1	9.2	13.0	
		Sensitivity	94.8	95.1	79.4	
		Specificity	39.9	86.6	83.6	
		AUROC	N/A	N/A	N/A	
MRI elastography	Various etiologies	Cutoff (kPa)	3.66	4.11	4.71	[15]
		Sensitivity	79	85	91	
		Specificity	81	85	81	
		AUROC	0.88	0.93	0.92	

Abbreviations: MRI, magnetic resonance imaging; HBV, hepatitis B virus; HCV, hepatitis C virus; NAFLD, nonalcoholic fatty liver disease; AUROC, area under the receiver operating characteristic; F, fibrosis stage; N/A, not available.

**Table 2:** Characteristics of elastography.

Elastography	Technique	Advantages	Disadvantages	References
US elastography	Static strain imaging	Real-time imaging with elastogram which can distinguish a tumor from background tissue.	Variability due to inconsistent compression (heartbeat). Semi-quantification	[16,17]
	1D transient elastography	The most widely used and validated.	Needs special equipment. Lacking B-mode	[10,11,18,19,20,21,22,23,24,25]
	Point shear wave elastography	Controllable ROI	Small ROI. Needs high-end US apparatus	[12,13]
	2D shear wave elastography	Controllable ROI. Real-time imaging	Needs high-end US apparatus	[14]
MRI elastography		Assessment of whole liver	Needs special equipment Not indicated to patients with claustrophobia	[26,27,28]

Abbreviation: US, ultrasound; B-mode, brightness-mode; ROI, region of interest; 1D, one dimensional; 2D, two dimensional.

**Table 3:** Serum tests and their diagnostic performances.

	Factors	Etiology		F2	F3	F4	Reference
FibroTest	α2-macroglobulin, haptoglobin, GGT, γ-globulin, total bilirubin, and apolipoprotein A1	HCV	AUROC	0.66	0.66	0.66	[30]
		HBV	AUROC	0.63	0.78	0.54	
		ALD	AUROC	0.65	0.66	0.82	
		NAFLD	AUROC	0.69	0.69	0.71	
APRI	AST, platelet count	HCV	Cutoff	0.7	1.0	2.0	[31]
			Sensitivity	77	61	46	
			Specificity	72	64	91	
			AUROC	0.77	0.80	0.83	
		HBV	Cutoff	0.5	1.0	1.5	[32]
			Sensitivity	70	50	36.9	
			Specificity	60	83	92.5	
			AUROC	0.72	0.76	0.72	
FIB-4 Index	Age, AST, ALT, platelet count	HCV	Cutoff		3.25		[33] (single study)
			Sensitivity		23		
			Specificity		97		
			AUROC		0.737		
		HBV	Cutoff	0.8–1.085	1.45–1.65	2.9–3.6	[34]
			Sensitivity	73	68	42	
			Specificity	62	75	96	
			AUROC	0.73	0.77	0.96	
M2BPGi		Various etiology	Cutoff	0.90–1.42	0.94–3.70	1.26–4.62	[35]
			Sensitivity	69	76	82	
			Specificity	78	76	84	
			AUROC	N/A	N/A	N/A	

Abbreviations: Ref, reference; GGT, gamma-glutamyl transferase; HCV, hepatitis C virus; HBV, hepatitis B virus; ALD, alcoholic liver disease; NAFLD, nonalcoholic fatty liver disease; AUROC, area under the receiver characteristic; F, fibrosis stage; APRI, aspartate transaminase to platelet ratio index; AST, aspartate aminotransferase; ALT, alanine aminotransferase; M2BPGi, Mac-2 binding protein glycosylation isomer; N/A, not available.

**Table 4:** Summary of matrix metalloproteinases (MMPs).

MMP Classification	Type	Aliases	Pathology	References
Collagenases	MMP-1	Interstitial collagenase	ECM degradation	[53,54,55]
	MMP-8	Neutrophil collagenase	Fibrosis attenuation	[56,57,58]
	MMP-13	Collagenase 3	Promote TGF-β1 activation	[59,60]
Gelatinases	MMP-2	Gelatinase A	Suppress collagen I expression	[61,62,63,64]
	MMP-9	Gelatinase B	Promote apoptosis of HSCs	[65,66,67,68]
Stromelysins	MMP-3	Stromelysin-1	ECM degradation. Activate pro-MMPs	[69,70,71]
	MMP-10	Stromelysin-2, Transin-2	Found in HCC	[72,73]
	MMP-11	Stomelysin-3	Tumor migration, invasion, metastasis	[74]
Matrilysins	MMP-7	Matrilysin-1, Pump-1, Uterine metalloproteinase	Activated in biliary atresia related liver fibrosis	[75,76,77]
	MMP-26	Matrilysin-2, Endometase	ECM degradation and activates MMP-9	[75,78,79]
Membranous Type	MMP-14	MT1-MMP	Angiogenesis and activates MMP-2	[80,81,82]
	MMP-15	MT2-MMP	Cell migration and invasion	[83]
	MMP-16	MT3-MMP	Cell invasion and metastases	[84]
	MMP-17	MT4-MMP	Expressed in breast cancer cells	[85]
	MMP-24	MT5-MMP	Brain specific	[86]
	MMP-25	MT6-MMP	Expressed in peripheral blood leukocytes	[87,88]
Others	MMP-12	Macrophage elastase	Macrophage migration	[89,90]
	MMP-19	RASI-1	Destruction and development of hepatic basement membrane	[91,92,93]
	MMP-20	Enamelysin	Degrades amelogenin	[94]
	MMP-22	N/A	Cloned from chicken fibroblast	[95]
	MMP-23	Femalysin	Expressed in reproductive tissues	[96,97]
	MMP-28	Epilysin	Degrades casein. Promotes EMT, migration and invasion of HCC cells.	[98,99,100,101]

Abbreviation: MMP, matrix metalloproteinase; MT, membrane type; ECM, extracellular matrix; TGF-β1, transforming growth factor-beta1; HSC, hepatic stellate cell; HCC, hepatocellular carcinoma; EMT, epithelial to mesenchymal transition; N/A, not available.

**Table 5:** Summary of tissue inhibitors of metalloproteinase.

TIMP Classification	Pathology	References
TIMP1	Inhibition of collagenase Inhibition of activation of pro-MMPs Inhibition of programmed cell death of HSCs	[104,105,106,107,108,109,110,111]
TIMP2	Inhibition of MT1-MMP, MMP-2 Activation of pro-MMP2	[112,113,114,115,116]
TIMP3	Promotion of apoptosis Regulation of inflammation through inhibition of ADAM17	[117]
TIMP4	Inhibition of MT1-MMP	[118]

Abbreviation: MMP, matrix metalloproteinase; HSC, hepatic stellate cell; MT. membrane type; ADAM17, a disintegrin and metalloproteinase 17.

## Abbreviations

HCC	Hepatocellular carcinoma
HBV	Hepatitis B virus
HCV	Hepatitis C virus
ALD	Alcoholic liver disease
NASH	Nonalcoholic liver disease
ECM	Extracellular matrix
HSC	Hepatic stellate cell
TGF-β1	Transforming growth factor beta 1
MMP	Matrix metalloproteinase
TIMP	Tissue inhibitor of metalloproteinase
SWE	Shear wave elastography
AUROC	Area under the receiver operating characteristics
ARFI	Acoustic radiation force impulse
ROI	Region of interest
2D	Two dimensional
MRE	Magnetic resonance elastography
NAFLD	Nonalcoholic fatty liver disease
FT	FibroTest
GGT	Gamma-glutamyl transferase
AST	Aspartate aminotransferase
APRI	Aspartate aminotransferase to platelet ratio index
AST	Aspartate aminotransferase
ULN	Upper limit of normal
APRICOT	Acquired immune deficiency syndrome Pegasys Ribabirin International Coinfection Trial
HIV	Human immunodeficiency virus
ALT	Alanine aminotransferase
M2MPGi	Mac-2 binding protein glycosylation isomer
TGF	Transforming growth factor
MT-MMP	Membranous type-matrix metalloproteinase
HBXIP	Hepatitis B virus X-interacting protein
SNPs	Single nucleotide polymorphisms
TNF	Tissue necrosis factor
IGF-1	Insulin-like growth factor-1
EMT	Epithelial to mesenchymal transition
ADAM17	A disintegrin and metalloproteinase

## References

[1] Masuzaki R., Yoshida H., Tateishi R., Shiina S., Omata M. (2008). Hepatocellular carcinoma in viral hepatitis: Improving standard therapy. Best Pract. Res. Clin. Gastroenterol..

[2] Bertuccio P., Turati F., Carioli G., Rodriguez T., La Vecchia C., Malvezzi M., Negri E. (2017). Global trends and predictions in hepatocellular carcinoma mortality. J. Hepatol..

[3] Omata M., Cheng A.L., Kokudo N., Kudo M., Lee J.M., Jia J., Tateishi R., Han K.H., Chawla Y.K., Shiina S. (2017). Asia–Pacific clinical practice guidelines on the management of hepatocellular carcinoma: A 2017 update. Hepatol. Int..

[4] Heimbach J.K., Kulik L.M., Finn R.S., Sirlin C.B., Abecassis M.M., Roberts L.R., Zhu A.X., Murad M.H., Marrero J.A. (2018). AASLD guidelines for the treatment of hepatocellular carcinoma. Hepatology.

[5] Galle P.R., Forner A., Llovet J.M., Mazzaferro V., Piscaglia F., Raoul J.L., Schirmacher P., Vilgrain V. (2018). EASL Clinical Practice Guidelines: Management of hepatocellular carcinoma. J. Hepatol..

[6] Kanda T., Goto T., Hirotsu Y., Masuzaki R., Moriyama M., Omata M. (2020). Molecular mechanisms: Connections between nonalcoholic fatty liver disease, steatohepatitis and hepatocellular carcinoma. Int. J. Mol. Sci..

[7] Yoshida H., Shiratori Y., Moriyama M., Arakawa Y., Ide T., Sata M., Inoue O., Yano M., Tanaka M., Fujiyama S. (1999). Interferon therapy reduces the risk for hepatocellular carcinoma: National surveillance program of cirrhotic and noncirrhotic patients with chronic hepatitis C in Japan. Ann. Intern. Med..

[8] Dienstag J.L. (2002). The role of liver biopsy in chronic hepatitis C. Hepatology.

[9] Masuzaki R., Zhao S.R., Csizmadia E., Yannas I., Karp S.J. (2013). Scar formation and lack of regeneration in adult and neonatal liver after stromal injury. Wound Repair Regen..

[10] Friedrich-Rust M., Ong M.F., Martens S., Sarrazin C., Bojunga J., Zeuzem S., Herrmann E. (2008). Performance of Transient Elastography for the Staging of Liver Fibrosis: A Meta-Analysis. Gastroenterology.

[11] Chon Y.E., Choi E.H., Song K.J., Park J.Y., Kim D.Y., Han K.H., Chon C.Y., Ahn S.H., Kim S.U. (2012). Performance of Transient Elastography for the Staging of Liver Fibrosis in Patients with Chronic Hepatitis B: A Meta-Analysis. PLoS ONE.

[12] Bota S., Herkner H., Sporea I., Salzl P., Sirli R., Neghina A.M., Peck-Radosavljevic M. (2013). Meta-analysis: ARFI elastography versus transient elastography for the evaluation of liver fibrosis. Liver Int..

[13] Lin Y., Li H., Jin C., Wang H., Jiang B. (2020). The diagnostic accuracy of liver fibrosis in non-viral liver diseases using acoustic radiation force impulse elastography: A systematic review and meta-analysis. PLoS ONE.

[14] Herrmann E., de Lédinghen V., Cassinotto C., Chu W.C.-W., Leung V.Y.-F., Ferraioli G., Filice C., Castera L., Vilgrain V., Ronot M. (2018). Assessment of biopsy-proven liver fibrosis by two-dimensional shear wave elastography: An individual patient data-based meta-analysis. Hepatology.

[15] Singh S., Venkatesh S.K., Wang Z., Miller F.H., Motosugi U., Low R.N., Hassanein T., Asbach P., Godfrey E.M., Yin M. (2015). Diagnostic performance of magnetic resonance elastography in staging liver fibrosis: A systematic review and meta-analysis of individual participant data. Clin. Gastroenterol. Hepatol..

[16] Lyshchik A., Higashi T., Asato R., Tanaka S., Ito J., Hiraoka M., Insana M.F., Brill A.B., Saga T., Togashi K. (2007). Cervical lymph node metastases: Diagnosis at sonoelastography—Initial experience. Radiology.

[17] Ko S.Y., Kim E.K., Sung J.M., Moon H.J., Kwak J.Y. (2014). Diagnostic performance of ultrasound and ultrasound elastography with respect to physician experience. Ultrasound Med. Biol..

[18] Masuzaki R., Tateishi R., Yoshida H., Sato T., Ohki T., Goto T., Yoshida H., Sato S., Sugioka Y., Ikeda H. (2007). Assessing liver tumor stiffness by transient elastography. Hepatol. Int..

[19] Sandrin L., Fourquet B., Hasquenoph J.M., Yon S., Fournier C., Mal F., Christidis C., Ziol M., Poulet B., Kazemi F. (2003). Transient elastography: A new noninvasive method for assessment of hepatic fibrosis. Ultrasound Med. Biol..

[20] Castéra L., Vergniol J., Foucher J., Le Bail B., Chanteloup E., Haaser M., Darriet M., Couzigou P., De Lédinghen V. (2005). Prospective comparison of transient elastography, Fibrotest, APRI, and liver biopsy for the assessment of fibrosis in chronic hepatitis C. Gastroenterology.

[21] De Lédinghen V., Hiriart J.B., Vergniol J., Merrouche W., Bedossa P., Paradis V. (2017). Controlled Attenuation Parameter (CAP) with the XL Probe of the Fibroscan^®^: A Comparative Study with the M Probe and Liver Biopsy. Dig. Dis. Sci..

[22] Masuzaki R., Tateishi R., Yoshida H., Goto E., Sato T., Ohki T., Imamura J., Goto T., Kanai F., Kato N. (2009). Prospective risk assessment for hepatocellular carcinoma development in patients with chronic hepatitis C by transient elastography. Hepatology.

[23] Fung J., Lai C.L., Seto W.K., Wong D.K.H., Yuen M.F. (2011). Prognostic significance of liver stiffness for hepatocellular carcinoma and mortality in HBeAg-negative chronic hepatitis B. J. Viral Hepat..

[24] Jung K.S., Kim S.U., Ahn S.H., Park Y.N., Kim D.Y., Park J.Y., Chon C.Y., Choi E.H., Han K.H. (2011). Risk assessment of hepatitis B virus-related hepatocellular carcinoma development using liver stiffness measurement (FibroScan). Hepatology.

[25] Robic M.A., Procopet B., Métivier S., Péron J.M., Selves J., Vinel J.P., Bureau C. (2011). Liver stiffness accurately predicts portal hypertension related complications in patients with chronic liver disease: A prospective study. J. Hepatol..

[26] Venkatesh S.K., Yin M., Ehman R.L. (2013). Magnetic resonance elastography of liver: Technique, analysis, and clinical applications. J. Magn. Reson. Imaging.

[27] Babu A.S., Wells M.L., Teytelboym O.M., Mackey J.E., Miller F.H., Yeh B.M., Ehman R.L., Venkatesh S.K. (2016). Elastography in chronic liver disease: Modalities, techniques, limitations, and future directions. Radiographics.

[28] Imbert-Bismut F., Ratziu V., Pieroni L., Charlotte F., Benhamou Y., Poynard T. (2001). Biochemical markers of liver fibrosis in patients with hepatitis C virus infection: A prospective study. Lancet.

[29] Sporea I., Gilja O.H., Bota S., Sirli R., Popescu A. (2013). Liver elastography—An update. Med. Ultrason..

[30] Poynard T., Morra R., Halfon P., Castera L., Ratziu V., Imbert-Bismut F., Naveau S., Thabut D., Lebrec D., Zoulim F. (2007). Meta-analyses of FibroTest diagnostic value in chronic liver disease. BMC Gastroenterol..

[31] Lin Z.H., Xin Y.N., Dong Q.J., Wang Q., Jiang X.J., Zhan S.H., Sun Y., Xuan S.Y. (2011). Performance of the aspartate aminotransferase-to-platelet ratio index for the staging of hepatitis C-related fibrosis: An updated meta-analysis. Hepatology.

[32] Xiao G., Yang J., Yan L. (2015). Comparison of diagnostic accuracy of aspartate aminotransferase to platelet ratio index and fibrosis-4 index for detecting liver fibrosis in adult patients with chronic hepatitis B virus infection: A systemic review and meta-analysis. Hepatology.

[33] Sterling R.K., Lissen E., Clumeck N., Sola R., Correa M.C., Montaner J., Sulkowski M.S., Torriani F.J., Dieterich D.T., Thomas D.L. (2006). Development of a simple noninvasive index to predict significant fibrosis in patients with HIV/HCV coinfection. Hepatology.

[34] Li Y., Chen Y., Zhao Y. (2014). The diagnostic value of the FIB-4 index for staging hepatitis B-related fibrosis: A meta-analysis. PLoS ONE.

[35] Ito K., Murotani K., Nakade Y., Inoue T., Nakao H., Sumida Y., Kamada Y., Yoneda M. (2017). Serum Wisteria floribunda agglutinin-positive Mac-2-binding protein levels and liver fibrosis: A meta-analysis. J. Gastroenterol. Hepatol..

[36] Wai C.T., Greenson J.K., Fontana R.J., Kalbfleisch J.D., Marrero J.A., Conjeevaram H.S., Lok A.S.F. (2003). A simple noninvasive index can predict both significant fibrosis and cirrhosis in patients with chronic hepatitis C. Hepatology.

[37] Ishiba H., Sumida Y., Tanaka S., Yoneda M., Hyogo H., Ono M., Fujii H., Eguchi Y., Suzuki Y., Yoneda M. (2018). The novel cutoff points for the FIB4 index categorized by age increase the diagnostic accuracy in NAFLD: A multi-center study. J. Gastroenterol..

[38] Shiratori Y., Imazeki F., Moriyama M., Yano M., Arakawa Y., Yokosuka O., Kuroki T., Nishiguchi S., Sata M., Yamada G. (2000). Histologic Improvement of Fibrosis in Patients with Hepatitis C Who Have Sustained Response to Interferon Therapy. Ann. Intern. Med..

[39] Minola E., Prati D., Suter F., Maggiolo F., Caprioli F., Sonzogni A., Fraquelli M., Paggi S., Conte D. (2002). Age at infection affects the long-term outcome of transfusion-associated chronic hepatitis C. Blood.

[40] Poynard T., Bedossa P., Opolon P. (1997). Natural history of liver fibrosis progression in patients with chronic hepatitis C. Lancet.

[41] Matsumura H., Moriyama M., Goto I., Tanaka N., Okubo H., Arakawa Y. (2000). Natural course of progression of liver fibrosis in Japanese patients with chronic liver disease type C—A study of 527 patients at one establishment. J. Viral Hepat..

[42] Masuzaki R., Tateishi R., Yoshida H., Arano T., Uchino K., Enooku K., Goto E., Nakagawa H., Asaoka Y., Kondo Y. (2012). Assessment of disease progression in patients with transfusion-associated chronic hepatitis C using transient elastography. World J. Gastroenterol..

[43] Somerville R.P.T., Oblander S.A., Apte S.S. (2003). Matrix metalloproteinases: Old dogs with new tricks. Genome Biol..

[44] Robert S., Gicquel T., Bodin A., Lagente V., Boichot E. (2016). Characterization of the MMP/TIMP Imbalance and Collagen Production Induced by IL-1β or TNF-α Release from Human Hepatic Stellate Cells. PLoS ONE.

[45] Hemmann S., Graf J., Roderfeld M., Roeb E. (2007). Expression of MMPs and TIMPs in liver fibrosis—A systematic review with special emphasis on anti-fibrotic strategies. J. Hepatol..

[46] Kawelke N., Vasel M., Sens C., von Au A., Dooley S., Nakchbandi I.A. (2011). Fibronectin protects from excessive liver fibrosis by modulating the availability of and responsiveness of stellate cells to active TGF-β. PLoS ONE.

[47] Shi F., Harman J., Fujiwara K., Sottile J. (2010). Collagen I matrix turnover is regulated by fibronectin polymerization. Am. J. Physiol. Cell Physiol..

[48] Altrock E., Sens C., Wuerfel C., Vasel M., Kawelke N., Dooley S., Sottile J., Nakchbandi I.A. (2015). Inhibition of fibronectin deposition improves experimental liver fibrosis. J. Hepatol..

[49] Arthur M.J.P. (2000). Fibrogenesis II. Metalloproteinases and their inhibitors in liver fibrosis. Am. J. Physiol. Gastrointest. Liver Physiol..

[50] Iredale J.P., Thompson A., Henderson N.C. (2013). Extracellular matrix degradation in liver fibrosis: Biochemistry and regulation. Biochim. Biophys. Acta.

[51] Visse R., Nagase H. (2003). Matrix metalloproteinases and tissue inhibitors of metalloproteinases: Structure, function, and biochemistry. Circ. Res..

[52] Naim A., Pan Q., Baig M.S. (2017). Matrix Metalloproteinases (MMPs) in Liver Diseases. J. Clin. Exp. Hepatol..

[53] Gross J., Lapiere C.M. (1962). Collagenolytic activity in amphibian tissues: A tissue culture assay. Proc. Natl. Acad. Sci. USA.

[54] Lichtinghagen R., Bahr M.J., Wehmeier M., Michels D., Haberkorn C.I., Arndt B., Flemming P., Manns M.P., Boeker K.H.W. (2003). Expression and coordinated regulation of matrix metalloproteinases in chronic hepatitis C and hepatitis C virus-induced liver cirrhosis. Clin. Sci. (Lond.).

[55] Iimuro Y., Nishio T., Morimoto T., Nitta T., Stefanovic B., Choi S.K., Brenner D.A., Yamaoka Y. (2003). Delivery of matrix metalloproteinase-1 attenuates established liver fibrosis in the rat. Gastroenterology.

[56] Harty M.W., Huddleston H.M., Papa E.F., Puthawala T., Tracy A.P., Ramm G.A., Gehring S., Gregory S.H., Tracy T.F. (2005). Repair after cholestatic liver injury correlates with neutrophil infiltration and matrix metalloproteinase 8 activity. Surgery.

[57] Siller-López F., Sandoval A., Salgado S., Salazar A., Bueno M., Garcia J., Vera J., Gálvez J., Hernández I., Ramos M. (2004). Treatment with Human Metalloproteinase-8 Gene Delivery Ameliorates Experimental Rat Liver Cirrhosis. Gastroenterology.

[58] Prystupa A., Boguszewska-Czubara A., Bojarska-Junak A., Toruń-Jurkowska A., Roliński J., Załuska W. (2015). Activity of MMP-2, MMP-8 and MMP-9 in serum as a marker of progression of alcoholic liver disease in people from Lublin Region, eastern Poland. Ann. Agric. Environ. Med..

[59] Hattori N., Mochizuki S., Kishi K., Nakajima T., Takaishi H., D’Armiento J., Okada Y. (2009). MMP-13 plays a role in keratinocyte migration, angiogenesis, and contraction in mouse skin wound healing. Am. J. Pathol..

[60] Prystupa A., Szpetnar M., Boguszewska-Czubara A., Grzybowski A., Grzybowski A., Sak J., Sak J., Załuska W. (2015). Activity of MMP1 and MMP13 and amino acid metabolism in patients with alcoholic liver cirrhosis. Med. Sci. Monit..

[61] Aimes R.T., Quigley J.P. (1995). Matrix metalloproteinase-2 is an interstitial collagenase. Inhibitor-free enzyme catalyzes the cleavage of collagen fibrils and soluble native type I collagen generating the specific 3/4 and 1/4 -length fragments. J. Biol. Chem..

[62] Patterson M.L., Atkinson S.J., Knäuper V., Murphy G. (2001). Specific collagenolysis by gelatinase A, MMP-2, is determined by the hemopexin domain and not the fibronectin-like domain. FEBS Lett..

[63] Radbill B.D., Gupta R., Ramirez M.C.M., DiFeo A., Martignetti J.A., Alvarez C.E., Friedman S.L., Narla G., Vrabie R., Bowles R. (2011). Loss of matrix metalloproteinase-2 amplifies murine toxin-induced liver fibrosis by upregulating collagen I expression. Dig. Dis. Sci..

[64] Onozuka I., Kakinuma S., Kamiya A., Miyoshi M., Sakamoto N., Kiyohashi K., Watanabe T., Funaoka Y., Ueyama M., Nakagawa M. (2011). Cholestatic liver fibrosis and toxin-induced fibrosis are exacerbated in matrix metalloproteinase-2 deficient mice. Biochem. Biophys. Res. Commun..

[65] Zhou X., Murphy F.R., Gehdu N., Zhang J., Iredale J.P., Benyon R.C. (2004). Engagement of alphavbeta3 integrin regulates proliferation and apoptosis of hepatic stellate cells. J. Biol. Chem..

[66] Roderfeld M., Rath T., Lammert F., Dierkes C., Graf J., Roeb E. (2010). Innovative immunohistochemistry identifies MMP-9 expressing macrophages at the invasive front of murine HCC. World J. Hepatol..

[67] Nart D., Yaman B., Yilmaz F., Zeytunlu M., Karasu Z., Kiliç M. (2010). Expression of matrix metalloproteinase-9 in predicting prognosis of hepatocellular carcinoma after liver transplantation. Liver Transpl..

[68] Hori T., Uemoto S., Walden L.B., Chen F., Baine A.-M.T., Hata T., Kogure T., Nguyen J.H. (2014). Matrix metalloproteinase-9 as a therapeutic target for the progression of fulminant liver failure with hepatic encephalopathy: A pilot study in mice. Hepatol. Res..

[69] Suzuki K., Morodomi T., Nagase H., Enghild J.J., Salvesen G. (1990). Mechanisms of Activation of Tissue Procollagenase by Matrix Metalloproteinase 3 (Stromelysin). Biochemistry.

[70] Bodey B., Bodey J.B., Siegel S.E., Kaiser H.E. (2000). Immunocytochemical detection of MMP-3 and -10 expression in hepatocellular carcinomas. Anticancer Res..

[71] Murawaki Y., Ikuta Y., Okamoto K., Koda M., Kawasaki H. (1999). Serum matrix metalloproteinase-3 (stromelysin-1) concentration in patients with chronic liver disease. J. Hepatol..

[72] Lefebvre O., Régnier C., Chenard M.P., Wendling C., Chambon P., Basset P., Rio M.C. (1995). Developmental expression of mouse stromelysin-3 mRNA. Development.

[73] Rouyer N., Wolf C., Chenard M.P., Rio M.C., Chambon P., Bellocq J.P., Basset P. (1994). Stromelysin-3 gene expression in human cancer: An overview. Invasion Metastasis.

[74] Wang B., Hsu C.J., Lee H.L., Chou C.H., Su C.M., Yang S.F., Tang C.H. (2018). Impact of matrix metalloproteinase-11 gene polymorphisms upon the development and progression of hepatocellular carcinoma. Int. J. Med. Sci..

[75] Uría J.A., López-Otín C. (2000). Matrilysin-2, a new matrix metalloproteinase expressed in human tumors and showing the minimal domain organization required for secretion, latency, and activity. Cancer Res..

[76] Park H.I., Ni J., Gerkema F.E., Liu D., Belozerov V.E., Sang Q.X. (2000). Identification and characterization of human endometase (Matrix metalloproteinase-26) from endometrial tumor. J. Biol. Chem..

[77] Huang C.-C., Chuang J.-H., Chou M.-H., Wu C.-L., Chen C.-M., Wang C.-C., Chen Y.-S., Chen C.-L., Tai M.-H. (2005). Matrilysin (MMP-7) is a major matrix metalloproteinase upregulated in biliary atresia-associated liver fibrosis. Mod. Pathol..

[78] Zeng Z.-S., Shu W.-P., Cohen A.M., Guillem J.G. (2002). Matrix metalloproteinase-7 expression in colorectal cancer liver metastases: Evidence for involvement of MMP-7 activation in human cancer metastases. Clin. Cancer Res..

[79] Zhao Y.-G., Xiao A.-Z., Newcomer R.G., Park H.I., Kang T., Chung L.W., Swanson M.G., Zhau H.E., Kurhanewicz J., Sang Q.-X.A. (2003). Activation of pro-gelatinase B by endometase/matrilysin-2 promotes invasion of human prostate cancer cells. J. Biol. Chem..

[80] Ohuchi E., Imai K., Fujii Y., Sato H., Seiki M., Okada Y. (1997). Membrane type 1 matrix metalloproteinase digests interstitial collagens and other extracellular matrix macromolecules. J. Biol. Chem..

[81] Harada T., Arii S., Mise M., Imamura T., Higashitsuji H., Furutani M., Niwano M., Ishigami S.I., Fukumoto M., Seiki M. (1998). Membrane-type matrix metalloproteinase-1(MT1-MMP) gene is overexpressed in highly invasive hepatocellular carcinomas. J. Hepatol..

[82] Pepper M.S. (2001). Extracellular proteolysis and angiogenesis. Thromb. Haemost..

[83] Zheng S., Wu H., Wang F., Lv J., Lu J., Fang Q., Wang F., Lu Y., Zhang S., Xu Y. (2019). The oncoprotein HBXIP facilitates metastasis of hepatocellular carcinoma cells by activation of MMP15 expression. Cancer Manag. Res..

[84] Scheau C., Badarau I.A., Costache R., Caruntu C., Mihai G.L., Didilescu A.C., Constantin C., Neagu M. (2019). The role of matrix metalloproteinases in the epithelial-mesenchymal transition of hepatocellular carcinoma. Anal. Cell. Pathol..

[85] Truong A., Yip C., Paye A., Blacher S., Munaut C., Deroanne C., Noel A., Sounni N.E. (2016). Dynamics of internalization and recycling of the prometastatic membrane type 4 matrix metalloproteinase (MT4-MMP) in breast cancer cells. FEBS J..

[86] Sekine-Aizawa Y., Hama E., Watanabe K., Tsubuki S., Kanai-Azuma M., Kanai Y., Arai H., Aizawa H., Iwata N., Takaomi C.S. (2001). Matrix metalloproteinase (MMP) system in brain: Identification and characterization of brain-specific MMP highly expressed in cerebellum. Eur. J. Neurosci..

[87] Pei D. (1999). Leukolysin/MMP25/MT6-MMP: A novel matrix metalloproteinase specifically expressed in the leukocyte lineage. Cell Res..

[88] Velasco G., Cal S., Merlos-Suárez A., Ferrando A.A., Alvarez S., Nakano A., Arribas J., López-Otín C. (2000). Human MT6-matrix metalloproteinase: Identification, progelatinase A activation, and expression in brain tumors. Cancer Res..

[89] Shapiro S.D., Kobayashi D.K., Ley T.J. (1993). Cloning and characterization of a unique elastolytic metalloproteinase produced by human alveolar macrophages. J. Biol. Chem..

[90] Shipley J.M., Wesselschmidt R.L., Kobayashi D.K., Ley T.J., Shapiro S.D. (1996). Metalloelastase is required for macrophage-mediated proteolysis and matrix invasion in mice. Proc. Natl. Acad. Sci. USA.

[91] Pendás A.M., Knäuper V., Puente X.S., Llano E., Mattei M.G., Apte S., Murphy G., López-Otín C. (1997). Identification and characterization of a novel human matrix metalloproteinase with unique structural characteristics, chromosomal location, and tissue distribution. J. Biol. Chem..

[92] Kolb C., Mauch S., Peter H.H., Krawinkel U., Sedlacek R. (1997). The matrix metalloproteinase RASI-1 is expressed in synovial blood vessels of a rheumatoid arthritis patient. Immunol. Lett..

[93] Jirouskova M., Zbodakova O., Gregor M., Chalupsky K., Sarnova L. (2012). Hepatoprotective Effect of MMP-19 Deficiency in a Mouse Model of Chronic Liver Fibrosis. PLoS ONE.

[94] Li W., Gibson C.W., Abrams W.R., Andrews D.W., DenBesten P.K. (2001). Reduced hydrolysis of amelogenin may result in X-linked amelogenesis imperfecta. Matrix Biol..

[95] Yang M., Kurkinen M. (1998). Cloning and characterization of a novel matrix metalloproteinase (MMP), CMMP, from chicken embryo fibroblasts. CMMP, Xenopus XMMP, and human MMP19 have a conserved unique cysteine in the catalytic domain. J. Biol. Chem..

[96] Velasco G., Pendás A.M., Fueyo A., Knäuper V., Murphy G., López-Otín C. (1999). Cloning and characterization of human MMP-23, a new matrix metalloproteinase predominantly expressed in reproductive tissues and lacking conserved domains in other family members. J. Biol. Chem..

[97] Pei D., Kang T., Qi H. (2000). Cysteine array matrix metalloproteinase (CA-MMP)/MMP-23 is a type II transmembrane matrix metalloproteinase regulated by a single cleavage for both secretion and activation. J. Biol. Chem..

[98] Lohi J., Wilson C.L., Roby J.D., Parks W.C. (2001). Epilysin, a novel human matrix metalloproteinase (MMP-28) expressed in testis and keratinocytes and in response to injury. J. Biol. Chem..

[99] Marchenko G.N., Strongin A.Y. (2001). MMP-28, a new human matrix metalloproteinase with an unusual cysteine-switch sequence is widely expressed in tumors. Gene.

[100] Saarialho-Kere U., Kerkelä E., Jahkola T., Suomela S., Keski-Oja J., Lohi J. (2002). Epilysin (MMP-28) expression is associated with cell proliferation during epithelial repair. J. Investig. Dermatol..

[101] Zhou J., Zheng X., Feng M., Mo Z., Shan Y., Wang Y., Jin J. (2019). Upregulated MMP28 in Hepatocellular Carcinoma Promotes Metastasis via Notch3 Signaling and Predicts Unfavorable Prognosis. Int. J. Biol. Sci.

[102] Fanjul-Fernández M., Folgueras A.R., Cabrera S., López-Otín C. (2010). Matrix metalloproteinases: Evolution, gene regulation and functional analysis in mouse models. Biochim. Biophys. Acta Mol. Cell Res..

[103] Allan J.A., Docherty A.J.P., Barker P.J., Huskisson N.S., Reynolds J.J., Murphy G. (1995). Binding of gelatinases A and B to type-I collagen and other matrix components. Biochem. J..

[104] El-Gindy I., El Rahman A.T.A., El-Alim M.A., Zaki S.S.A. (2003). Diagnostic potential of serum matrix metalloproteinase-2 and tissue inhibitor of metalloproteinase-1 as non-invasive markers of hepatic fibrosis in patients with HCV related chronic liver disease. Egypt. J. Immunol..

[105] Yata Y., Takahara T., Furui K., Zhang L.P., Jin B., Watanabe A. (1999). Spatial distribution of tissue inhibitor of metalloproteinase-1 mRNA in chronic liver disease. J. Hepatol..

[106] Ruiz V., Ordóñez R.M., Berumen J., Ramírez R., Uhal B., Becerril C., Pardo A., Selman M. (2003). Unbalanced collagenases/TIMP-1 expression and epithelial apoptosis in experimental lung fibrosis. Am. J. Physiol. Lung Cell. Mol. Physiol..

[107] Selman M., Ruiz V., Cabrera S., Segura L., Ramírez R., Barrios R., Pardo A. (2000). TIMP-1, -2, -3, and -4 in idiopathic pulmonary fibrosis. A prevailing nondegradative lung microenvironment?. Am. J. Physiol. Lung Cell. Mol. Physiol..

[108] Johnson T.S., Haylor J.L., Thomas G.L., Fisher M., El Nahas A.M. (2002). Matrix metalloproteinases and their inhibitions in experimental renal scarring. Exp. Nephrol..

[109] Hörstrup J.H., Gehrmann M., Schneider B., Plöger A., Froese P., Schirop T., Kampf D., Frei U., Neumann R., Eckardt K.-U. (2002). Elevation of serum and urine levels of TIMP-1 and tenascin in patients with renal disease. Nephrol. Dial. Transpl..

[110] Phillips P.A., McCarroll J.A., Park S., Wu M.J., Pirola R., Korsten M., Wilson J.S., Apte M.V. (2003). Rat pancreatic stellate cells secrete matrix metalloproteinases: Implications for extracellular matrix turnover. Gut.

[111] Ishihara T., Hayasaka A., Yamaguchi T., Kondo F., Saisho H. (1998). Immunohistochemical study of transforming growth factor-beta 1, matrix metalloproteinase-2,9, tissue inhibitors of metalloproteinase-1,2, and basement membrane components at pancreatic ducts in chronic pancreatitis. Pancreas.

[112] Böker K.H., Pehle B., Steinmetz C., Breitenstein K., Bahr M., Lichtinghagen R. (2000). Tissue inhibitors of metalloproteinases in liver and serum/plasma in chronic active hepatitis C and HCV-induced cirrhosis. Hepatogastroenterology.

[113] Lichtinghagen R., Michels D., Haberkorn C.I., Arndt B., Bahr M., Flemming P., Manns M.P., Boeker K.H.W. (2001). Matrix metalloproteinase (MMP)-2, MMP-7, and tissue inhibitor of metalloproteinase-1 are closely related to the fibroproliferative process in the liver during chronic hepatitis C. J. Hepatol..

[114] Kossakowska A.E., Edwards D.R., Lee S.S., Urbanski L.S., Stabbler A.L., Zhang C.L., Phillips B.W., Zhang Y., Urbanski S.J. (1998). Altered balance between matrix metalloproteinases and their inhibitors in experimental biliary fibrosis. Am. J. Pathol..

[115] Wang Z., Juttermann R., Soloway P.D. (2000). TIMP-2 is required for efficient activation of proMMP-2 in vivo. J. Biol. Chem..

[116] Kandalam V., Basu R., Abraham T., Wang X., Soloway P.D., Jaworski D.M., Oudit G.Y., Kassiri Z. (2010). TIMP2 Deficiency Accelerates Adverse Post–Myocardial Infarction Remodeling Because of Enhanced MT1-MMP Activity Despite Lack of MMP2 Activation. Circ. Res..

[117] Mohammed F.F., Smookler D.S., Taylor S.E.M., Fingleton B., Kassiri Z., Sanchez O.H., English J.L., Matrisian L.M., Au B., Yeh W.C. (2004). Abnormal TNF activity in Timp3-/- mice leads to chronic hepatic inflammation and failure of liver regeneration. Nat. Genet..

[118] Takawale A., Fan D., Basu R., Shen M., Parajuli N., Wang W., Wang X., Oudit G.Y., Kassiri Z. (2014). Myocardial recovery from ischemia-reperfusion is compromised in the absence of tissue inhibitor of metalloproteinase 4. Circ. Heart Fail..

[119] Nakchbandi I.A., van der Merwe S.W. (2009). Current understanding of osteoporosis associated with liver disease. Nat. Rev. Gastroenterol. Hepatol..

[120] Kawelke N., Bentmann A., Hackl N., Hager H.D., Feick P., Geursen A., Singer M.V., Nakchbandi I.A. (2008). Isoform of fibronectin mediates bone loss in patients with primary biliary cirrhosis by suppressing bone formation. J. Bone Miner. Res..

[121] Hackl N.J., Bersch C., Feick P., Antoni C., Franke A., Singer M.V., Nakchbandi I.A. (2010). Circulating fibronectin isoforms predict the degree of fibrosis in chronic hepatitis C. Scand. J. Gastroenterol..

[122] Kuno A., Ikehara Y., Tanaka Y., Ito K., Matsuda A., Sekiya S., Hige S., Sakamoto M., Kage M., Mizokami M. (2013). A serum “sweet-doughnut” protein facilitates fibrosis evaluation and therapy assessment in patients with viral hepatitis. Sci. Rep..

[123] Narimatsu H. (2015). Development of M2BPGi: A novel fibrosis serum glyco-biomarker for chronic hepatitis/cirrhosis diagnostics. Expert Rev. Proteom..

[124] Baudi I., Inoue T., Tanaka Y. (2020). Novel biomarkers of hepatitis B and hepatocellular carcinoma: Clinical significance of HBcrAg and M2BPGI. Int. J. Mol. Sci..

[125] Nishikawa H., Enomoto H., Iwata Y., Kishino K., Shimono Y., Hasegawa K., Nakano C., Takata R., Nishimura T., Yoh K. (2016). Serum Wisteria floribunda agglutinin-positive Mac-2-binding protein for patients with chronic hepatitis B and C: A comparative study. J. Viral Hepat..

